# Investigating the mechanisms linking vitamin D to coronary artery disease: A mediating proteomics Mendelian randomisation study

**DOI:** 10.1097/MD.0000000000049798

**Published:** 2026-07-17

**Authors:** Yanqiong Xue, Shunhua Cao, Linxia Zhang, Jiaquan Chen, Aili Ning

**Affiliations:** a Department of Cardiology, Yiyang Central Hospital, Medical Education Center of Jinan University, Yiyang, Hunan, China; b Department of Geriatrics, Yiyang Central Hospital, Medical Education Center of Jinan University, Yiyang, Hunan, China.

**Keywords:** causal relationship, coronary artery disease, mediation analysis, plasma proteomics, vitamin D

## Abstract

Coronary artery disease (CAD) is a leading cause of mortality and morbidity globally, with its elevated rates of disability and death posing a significant public health concern. Vitamin D is a crucial bioactive compound involved in numerous physiological processes and has garnered considerable interest due to its potential health benefits. The association between vitamin D and CAD has been a prominent focus of scholarly investigation. However, there remains considerable debate regarding whether vitamin D confers protective effects against CAD, and the underlying mechanisms by which vitamin D influences CAD remain inadequately understood. Mendelian randomization analysis was performed using large-scale genome-wide association study data to examine the causal relationship between serum 25-hydroxyvitamin D (25(OH)D) levels and CAD. Plasma proteomics data were subsequently employed for mediation analysis, followed by enrichment analysis to identify intermediary metabolic or signaling pathways through which serum 25(OH)D may mediate the onset and progression of CAD. The Mendelian randomization analysis indicated that higher serum 25(OH)D levels were associated with a reduced risk of CAD (odds ratio [95% confidence interval]: 0.799 [0.643–0.993], *P* = .043). No evidence of pleiotropy (*P* = .949) or heterogeneity (*P* = .630) was observed in the results. The protein-mediated analysis identified 19 plasma proteins, including Serine/threonine-protein kinase TBK1, membrane associating domain domain-containing protein 2, and interleukin-17D, as key mediators through which reduced vitamin D levels contribute to the development of CAD. The mediation effects ranged from 4.85 to 34.49%. Following the identification of these 19 mediating proteins, 59 intermediary pathways were further pinpointed through which serum vitamin D influences CAD risk. Increased levels of 25(OH)D may reduce the risk of CAD. Further, plasma proteomics-mediated analyses have uncovered potential mechanisms through which 25(OH)D influences the development of CAD, offering a detailed framework for understanding the relationship between vitamin D deficiency and CAD progression. This provides novel evidence to support the recommendation of appropriate vitamin D supplementation as part of lifestyle guidance for CAD patients.

## 1. Introduction

Cardiovascular disease (CVD) is the leading cause of death globally, posing a significant threat to human health and placing a substantial burden on healthcare systems. According to the World Health Organization, an estimated 17.9 million individuals died from CVDs in 2019, accounting for 32% of all global deaths. The Global Burden of Disease Study projects that by 2030, the number of deaths attributable to CVDs will rise to 24 million.^[1]^ Coronary artery disease (CAD) is the most common and threatening component of CVD, characterized by narrowing or blockage of blood vessels due to coronary atherosclerosis, and in severe cases it can lead to life-threatening events such as angina pectoris and myocardial infarction.^[2]^ Consequently, identifying new risk factors for CAD that may have potential therapeutic implications is of paramount importance.

Vitamin D is a fat-soluble vitamin that is mainly synthesized in the skin through ultraviolet light exposure and dietary supplementation.^[3]^ Notably, vitamin D deficiency is highly prevalent, affecting approximately 30 to 50% of the global population, and is observed across all ethnicities and age groups.^[4]^ The conversion of vitamin D to 25-hydroxyvitamin D (25(OH)D) is catalyzed by the enzyme 25-hydroxylase. It is broadly recognized that the concentration of 25(OH)D serves as a reliable indicator of the body’s vitamin D status.^[5]^ In recent years, the relationship between vitamin D deficiency and the risk of CAD has received increasing attention. Several observational studies, as well as meta-analyses, have found that low 25(OH)D levels are negatively associated with the development of CAD.^[6,7]^ In addition, several studies have found that vitamin D deficiency is associated with more diffuse CAD and greater severity of coronary artery stenosis. This suggests that low vitamin D levels may be linked not only to an increased risk of CAD but also to its severity.^[8,9]^ However, a clinical study of 5108 participants did not find that vitamin D supplementation could prevent the development of CAD.^[10]^ Such inconsistency further complicates the understanding of the relationship between serum vitamin D and CAD.

Mendelian randomization (MR) is an epidemiological research method that explores the causal relationship between exposure and outcome using genetic variation as the instrumental variable.^[11]^ This method has gained increasing popularity due to its ability to avoid reverse causality and mitigate confounding biases.^[12]^ Plasma proteins play a critical role in mediating the development of various diseases.^[13]^ In recent years, as genome-wide association study (GWAS) data of plasma proteomics have been reported, it has opened up the possibility to utilize MR to delve deeper into the mechanisms behind causality.^[14]^ In the present study, based on large-scale GWAS data, the causal relationship between serum 25(OH)D levels and CAD was systematically explored using MR methods, and plasma proteomics were further mediated to delve deeper into the mechanisms by which 25(OH)D may contribute to the development of CAD. The present findings may facilitate the discovery of novel preventive strategies as well as intervention targets for CAD.

## 2. Methods

### 2.1. Data sources

Data associating serum 25(OH)D levels were derived from an expanded GWAS dataset provided by the Study of Underlying Genetic Determinants of Vitamin D and Highly Related Traits Consortium, published by Jiang et al in 2018.^[15]^ The database comprises summary-level genome-wide association data linking single nucleotide polymorphism (SNP) genotypes with serum 25(OH)D levels from 79,366 participants of European ancestry across 31 cohorts. As previously outlined in the literature, each study included in the database received approval from the relevant human research ethics committees, and all participants provided informed consent. The GWAS data related to CAD incidence were obtained from the FinnGen project. FinnGen is a large-scale genomic initiative that analyzes over 500,000 samples from the Finnish biobank, associating genetic variations with health data to enhance the understanding of disease mechanisms and susceptibility. This project is a collaborative effort involving Finnish research institutions, biobanks, and international industry partners.^[16]^ The R10 version of the GWAS data was released on May 11, 2023. Plasma proteome-based GWAS data were published by Sun et al in 2018,^[17]^ including approximately 50,000 participants from a randomized trial, with varying blood donation intervals. Between mid-2012 and mid-2014, 25 centers of the United Kingdom National Health Service Blood and Transplant service recruited participants aged 18 or older. A multiplexed aptamer-based technique (SOMAscan assay) was employed to measure the relative concentrations of 3282 plasma proteins or protein complexes, using 4034 modified aptamers for quantification. All datasets included European populations, comprising both male and female participants. Detailed information about these datasets can be found in Table 1 and Table S1, Supplemental Digital Content 1. Ethical approval was not applicable for this study because it was based on publicly available summary-level GWAS data. No individual-level data were used, and no new samples were collected or measured. Therefore, informed consent from participants was not applicable.

**Table 1:** Information of datasets included in the study.

Trait	Case	Sample size	Year	Author	Sex	Population	nSNP
Vitamin D levels	79,366	79,366	2018	Jiang X	Males and Females	European	2,538,249
Major coronary heart disease event	46,959	412,181	2023	FINNGEN	Males and Females	European	20,167,370
Plasma proteome	3301	3301	2018	Sun BB	Males and Females	European	10,534,735

Trait: phenotype of the dataset.

nSNP = number of available single nucleotide polymorphism.

### 2.2. Definition of CAD

CAD was initially identified using the primary diagnosis database for hospitalized patients, with international classification of diseases (ICD)-10 codes “I20.0, I21, I22” or ICD-9 codes “410, 4110.” The diagnosis based on ICD-10 codes during hospitalization was made using multiple methods, including clinical symptoms, electrocardiographic examination, imaging techniques (such as coronary angiography and coronary computed tomography angiography), and biochemical markers. Based on the information from the data set sources, there is no sample overlap in the MR analysis of this study.

### 2.3. MR analysis

**Figure 1.**
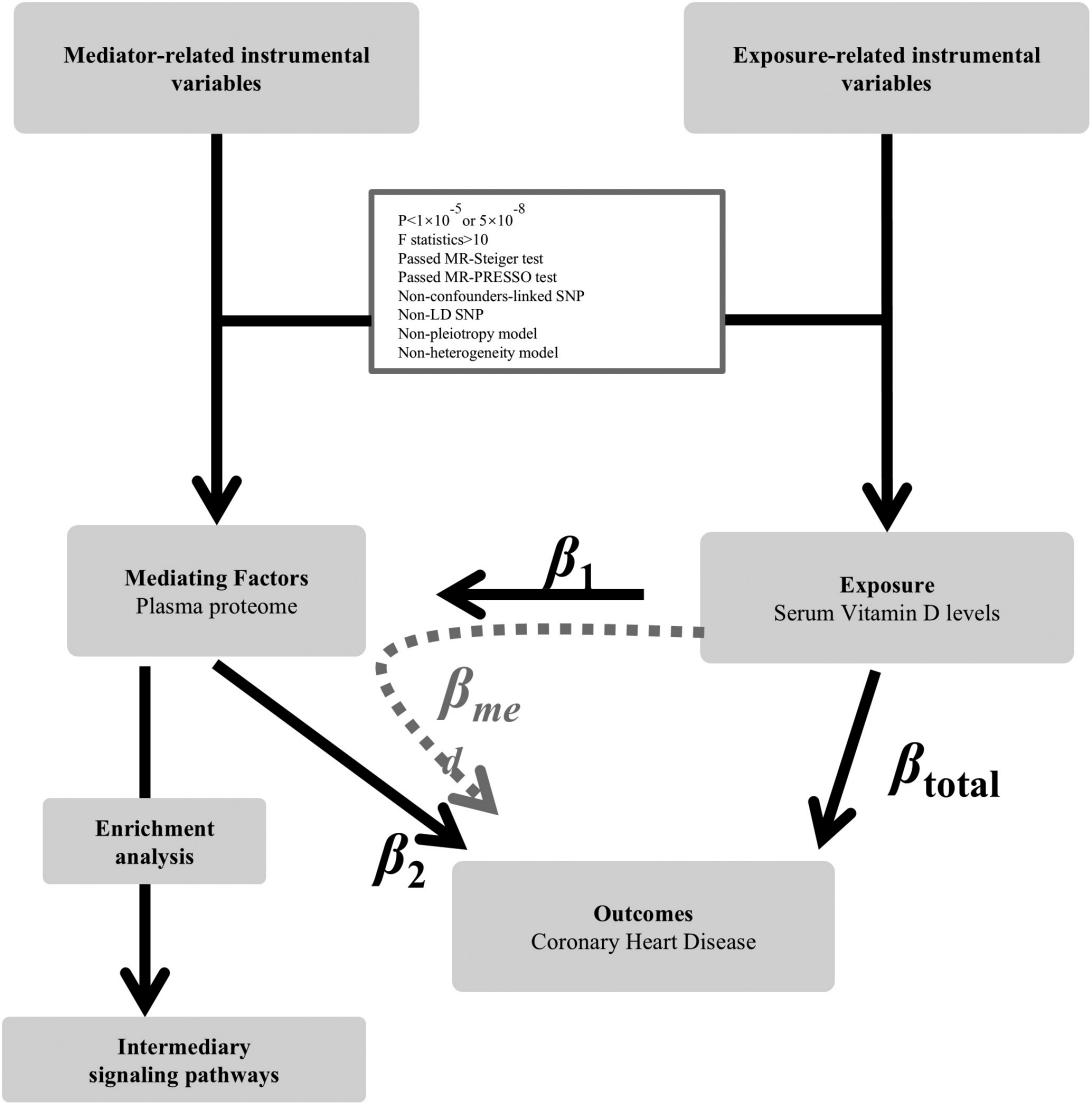
MR analysis diagram. LD = linkage disequilibrium, MR = Mendelian randomization, SNP = single nucleotide polymorphism.

The study design is illustrated in Figure 1. To meet the necessary assumptions for instrumental variables, including relevance, independence, and exclusion restriction, SNPs associated with risk factors were first selected at a genome-wide significance level (*P* < 5 × 10^−8^). For mediation MR analysis, given that the associations between plasma proteins and genetic variants may vary, extracting an excessive number of SNPs could increase heterogeneity, while extracting too few SNPs might limit the variance explained. As a result, SNP extraction was conducted at a more relaxed genome-wide potential significance threshold (*P* < 1 × 10^−5^). To address the issue of multicollinearity caused by multiple SNPs capturing the same effect, SNPs in linkage disequilibrium were removed based on an *R*^2^ threshold < 0.001 and a window size > 10,000 kb. SNP data associated with the outcomes were extracted from the results dataset based on SNP identifiers. During this process, ambiguous and palindromic SNPs were excluded, and SNPs were matched between the 2 datasets. The *F*-statistic for each SNP was subsequently calculated.^[18]^ SNPs with an *F*-statistic < 10 were considered weak instrumental variables, indicating insufficient strength of association with the risk factor, and were thus removed. Mendelian Randomization Pleiotropy RESidual Sum and Outlier testing was conducted to identify and exclude potentially pleiotropic SNPs. The MR-Steiger test was employed to assess the causal direction of each SNP, and those with an incorrect causal direction were excluded.^[19]^ Following stringent filtering, the remaining SNPs were deemed valid instrumental variables.

Various robust MR methods were employed to estimate the causal relationship between exposure and outcome, based on a range of underlying assumptions. These included inverse variance weighting (IVW), MR-Egger regression, weighted median (WM), weighted mode, and MR-Robust Adjusted Profile Score. IVW is among the most commonly utilized approaches in MR analysis. It integrates SNP ratio estimates with IVW within a meta-analytic framework to quantify the effect of risk factors on outcomes.^[20]^ This method was the primary approach used in the present analysis and was the main method employed in the present study. Similar to IVW, MR-Egger regression employs a regression framework, but includes an intercept term that allows for the assessment of horizontal pleiotropy. MR-Egger regression is particularly favored when evidence of pleiotropy is present. In comparison to both IVW and MR-Egger methods, the WM method demonstrates enhanced robustness, making it especially reliable in situations where such variation must be accounted for. Furthermore, this study incorporated the recently developed MR-Robust Adjusted Profile Score method, which utilizes a random effects distribution to directly address the pleiotropic effects of genetic variation. This innovative approach provides greater robustness than traditional MR techniques. Statistical significance of the final results was determined using a threshold of *P* < .05. By combining multiple robust MR methods, this comprehensive approach ensures reliable and unbiased estimates of causal effects, even in the presence of complex genetic structures or invalid instruments.

The MR-Egger method was used to estimate the magnitude of horizontal pleiotropy. When the *P* value for the MR-Egger intercept was < .05, it was considered that the instrumental variables were severely affected by horizontal pleiotropy, and the results were deemed unreliable. In the presence of pleiotropy, MR-Egger regression was used as the primary analysis method. Cochran *Q* test was applied, with a *P* value < .05 indicating the presence of heterogeneity. In the event of heterogeneity, the IVW random effects model was employed, using the WM method as a reference. In the absence of heterogeneity, the IVW random effects model served as the primary analysis method.^[21]^ Further, an overall MR-Steiger test was conducted to ensure the correct causal direction. Statistical power was also calculated to assess the reliability of negative results.^[22]^

### 2.4. Mediation and enrichment analysis

A 2-step MR analysis was conducted using GWAS summary data to assess whether plasma proteins mediate the relationship between elevated serum 25(OH)D levels and the risk of CAD. In the first step, a 2-sample MR analysis was performed to evaluate the association between serum 25(OH)D levels and the plasma proteome. In the second step, a separate 2-sampleMR analysis was conducted to investigate the relationship between the plasma proteome and CAD incidence. Proteins that were significant in all 3 analyses were considered to demonstrate partial mediation, while proteins significant only in the 2-step mediation analysis were interpreted as exhibiting complete mediation. Proteins that were not significant in the 2-step mediation analysis were regarded as not mediating the association. The indirect effect was calculated using the formula *β*_1_ × *β*_2_, with the direct effect derived by subtracting the indirect effect from the total effect. To explore enriched pathways associated with the mediating plasma proteins, the Reactome knowledgebase was employed.^[23]^ Reactome is a peer-reviewed human biological pathway and reaction database. Overrepresentation analysis was performed to determine whether specific Reactome pathways were enriched in the gene list, generating probability scores and significance *P* values.

### 2.5. Statistical software

All statistical analyses in the present study were conducted using R (version 4.2.3) and the R packages “TwoSampleMR,” “MR-PRESSO,” and “mr.raps.”

## 3. Results

### 3.1. Dataset information

**Figure 2.**
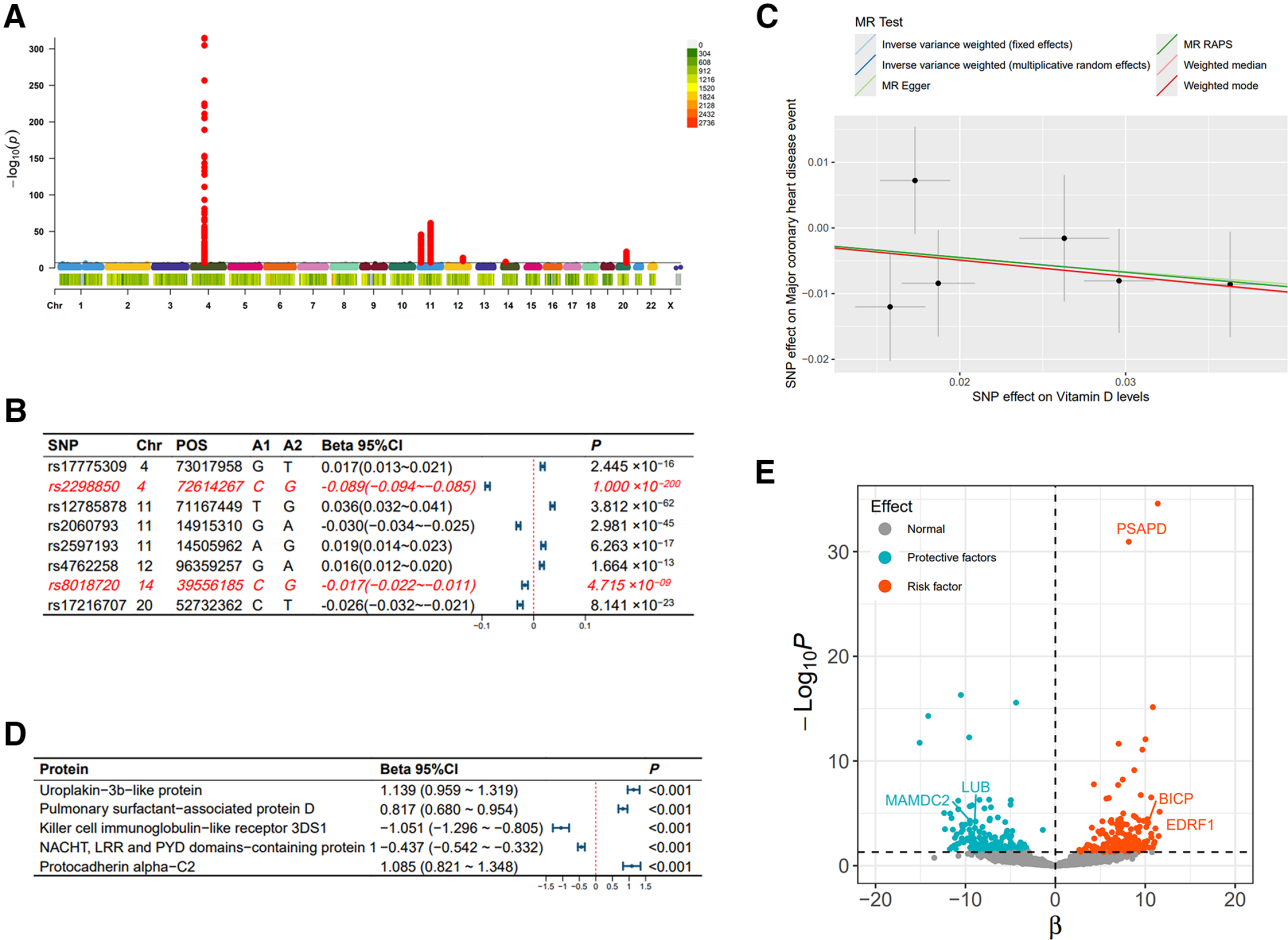
Exposure-related analysis diagram. (A) The Manhattan plot summarizes the results of the GWAS for serum vitamin D levels. (B) SNPs that are genome-wide significant for serum vitamin D levels, with red highlighting indicating invalid instrumental variables. (C) Causal relationship between serum vitamin D levels and CAD incidence, with the regression line representing the causal effect. (D) The top 5 plasma proteins most influenced by serum vitamin D levels. (E) A scatter plot showing the significance levels of the effect of serum vitamin D levels on plasma proteins. CAD = coronary artery disease, CI = confidence interval, GWAS = genome-wide association study, MR = Mendelian randomization, RAPS = Robust Adjusted Profile Score, SNP = single nucleotide polymorphism.

**Figure 3.**
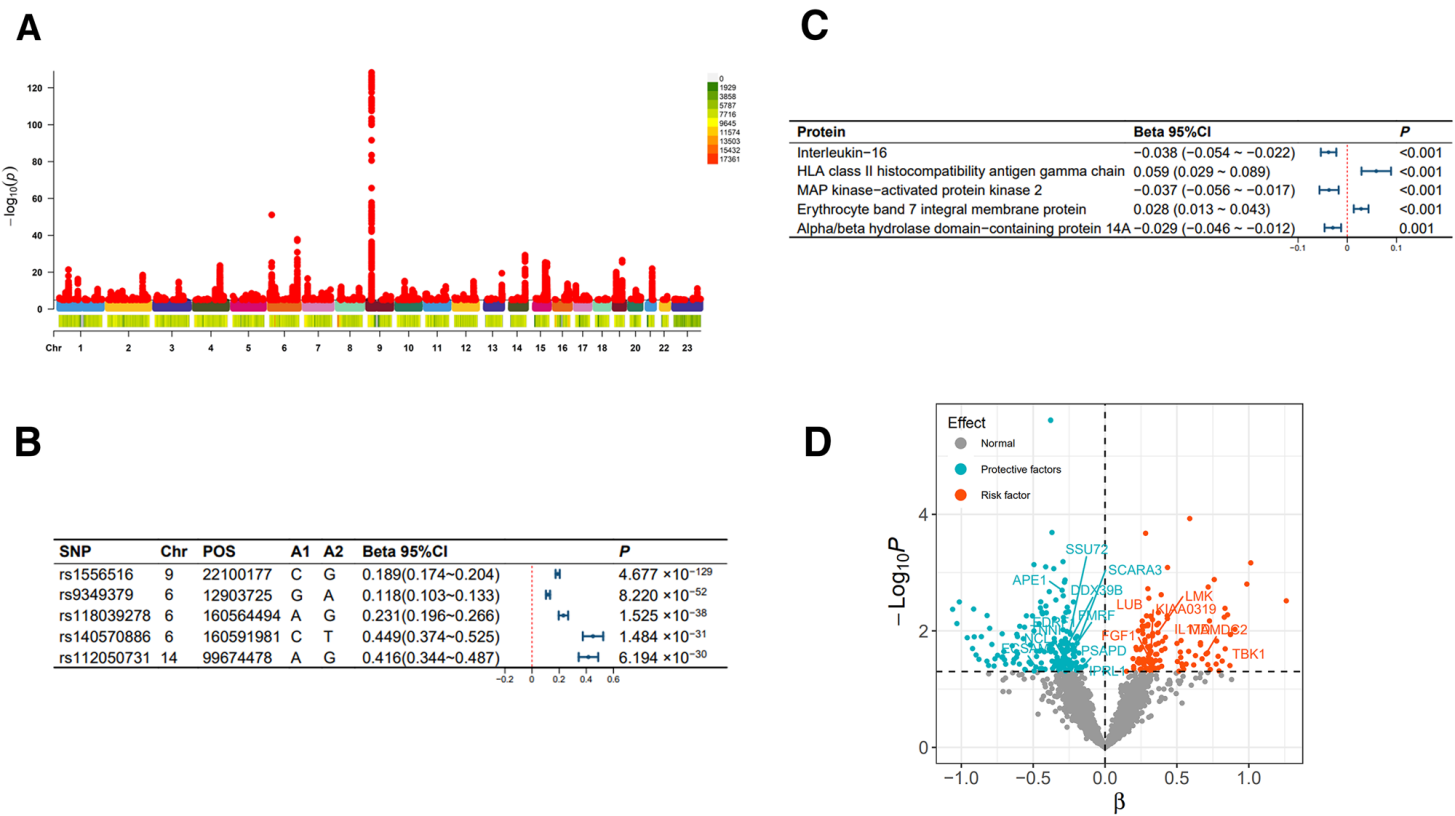
Outcome-related analysis diagram. (A) The Manhattan plot summarizes the results of the GWAS for CAD incidence. (B) The top 5 SNPs most significantly associated with CAD incidence. (C) The top 5 plasma proteins most significantly influencing CAD incidence. (D) A scatter plot showing the significance levels of the effect of plasma proteins on CAD incidence, with specified proteins indicating mediation effects. CAD = coronary artery disease, Chr = chromosome, CI = confidence interval, GWAS = genome-wide association study, HLA = human leukocyte antigen, MAP = mitogen-activated protein, POS = position in base pairs, SNP = single nucleotide polymorphism.

The SNPs associated with 25(OH)D levels predominantly originated from chromosomes 4, 11, 12, 14, and 20 (Fig. 2A). After exclusion of LD, the SNPs that reached genome-wide significance were rs17775309, rs2298850, rs12785878, rs2060793, rs2597193, rs4762258, rs8018720, and rs17216707 (Fig. 2B). The SNPs associated with CAD incidence were derived from multiple chromosomes (Fig. 3A), with rs1556516 identified as the top SNP (Fig. 3B).

### 3.2. Instrumental variable selection

In the MR analysis, a total of 8 SNPs associated with 25(OH)D levels were initially screened. Of these, 2 were identified as palindromic or ambiguous SNPs (rs2298850, rs8018720), and no SNPs were found to exhibit pleiotropy or incorrect causal direction. Consequently, 6 eligible SNPs were retained for the study. In the mediation MR analysis, 131,348 SNPs related to the exposure were initially screened, with no weak instrumental variables identified. In the outcome dataset, 8567 SNPs were excluded due to missing data. Additionally, 21,050 palindromic or ambiguous SNPs were removed during the data merging process. The MR-Steiger test revealed no SNPs with incorrect causal direction. Following Bonferroni correction, 2232 SNPs directly associated with the outcome were excluded. Ultimately, 99,499 eligible SNPs were included in the analysis. Information regarding the SNPs included in the MR analysis is provided in Table S2, Supplemental Digital Content 2.

### 3.3. MR analysis

**Figure 4.**
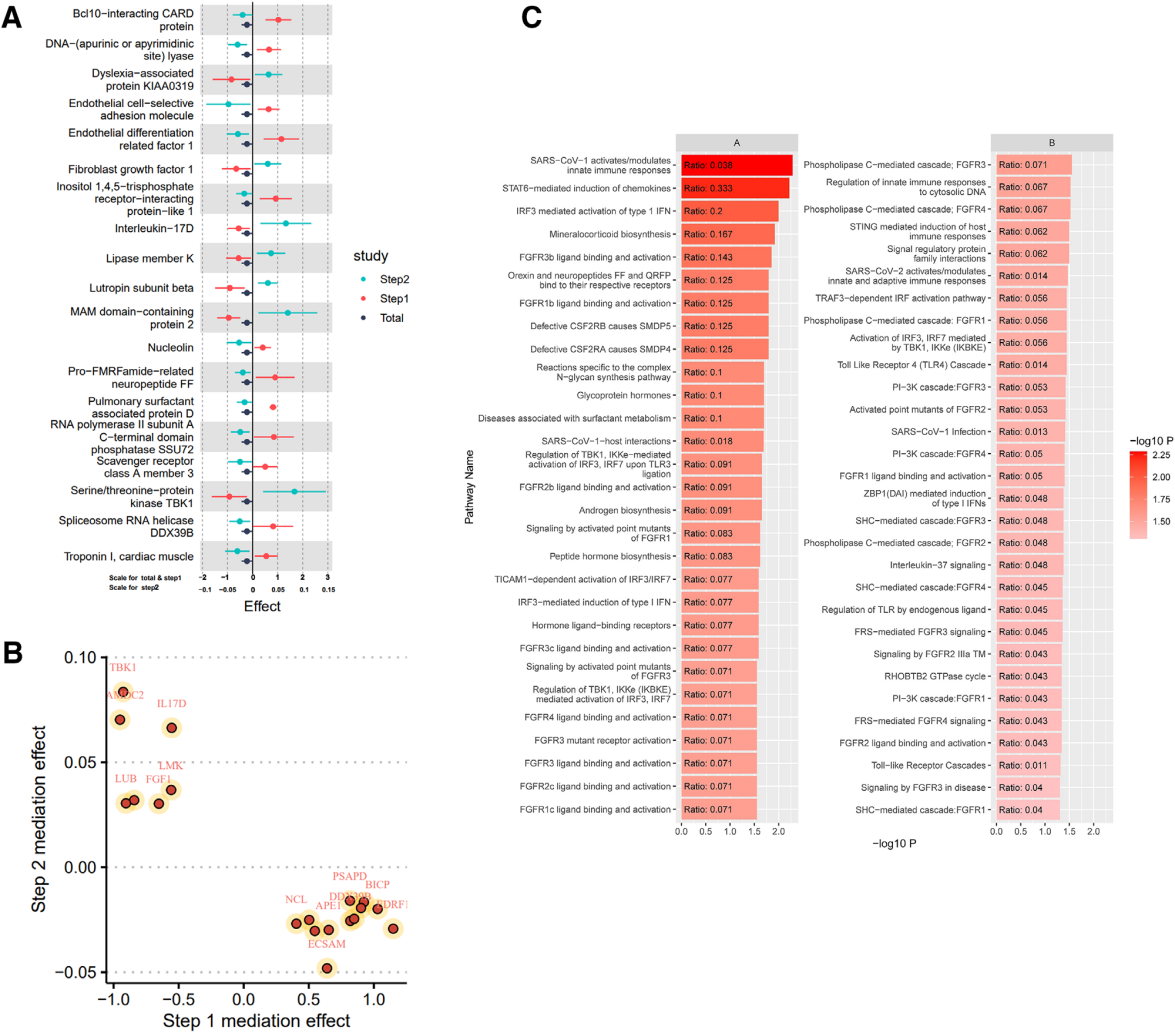
Mediation and enrichment analysis results. (A) Forest plot for the 2-step mediation effect of proteins significantly affecting the relationship between serum vitamin D levels and CAD incidence. (B) Scatter plot showing the 2-step mediation effect of proteins significantly affecting the relationship between serum vitamin D levels and CAD incidence. (C) Metabolic or signaling pathways significantly influenced by serum vitamin D levels and associated with CAD incidence. CAD = coronary artery disease.

In the MR analysis, it was found that higher 25(OH)D levels were associated with a reduced risk of CAD (odds ratio [95% confidence interval]: 0.799 [0.643–0.993], *P* = .043) (Table 2, Fig. 2C). The results showed no evidence of pleiotropy (*P* = .949) or heterogeneity (*P* = .630). In the mediation MR analysis, it was first identified that higher 25(OH)D levels were associated with the modulation of 378 plasma protein levels (Fig. 2D and 2E). Among these, 301 proteins were found to have causal effects on CAD incidence (Fig. 3C and 3D). Ultimately, 19 plasma proteins were identified that mediated the protective effect of elevated 25(OH)D levels on CAD risk, with mediation effects ranging from 4.85 to 34.49% (Table 2, Fig. 4A and 4B). Specifically, the 3 plasma proteins with the strongest mediation effects were Serine/threonine-protein kinase TBK1, membrane associating domain (MAM) domain-containing protein 2, and interleukin (IL)-17D. The MR analysis results for all batches are provided in Table S3, Supplemental Digital Content 3, with sensitivity analysis results available in Tables S4–S6, Supplemental Digital Content 4. The effect information for mediating proteins is listed in Table S7, Supplemental Digital Content 5.

**Table 2:** Mediating protein results and mediation effects.

Mediator	XY		XM		MY		Mediating direction	Mediating effect	Mediating ratio
	OR 95% CI	*P*	OR 95% CI	*P*	OR 95% CI	*P*			
Serine/threonine-protein kinase TBK1	0.799 (0.643–0.993)	.043	0.396 (0.196–0.799)	.010	1.087 (1.021–1.157)	.020	TRUE	Partial mediation	34.49%
MAM domain-containing protein 2	0.799 (0.643–0.993)	.043	0.386 (0.244–0.612)	< .001	1.073 (1.012–1.138)	.025	TRUE	Partial mediation	29.79%
Interleukin-17D	0.799 (0.643–0.993)	.043	0.575 (0.367–0.900)	.015	1.069 (1.016–1.124)	.017	TRUE	Partial mediation	16.38%
Endothelial differentiation-related factor 1	0.799 (0.643–0.993)	.043	3.154 (1.553–6.406)	.001	0.971 (0.950–0.993)	.010	TRUE	Partial mediation	14.99%
Endothelial cell-selective adhesion molecule	0.799 (0.643–0.993)	.043	1.897 (1.229–2.929)	.004	0.953 (0.912–0.996)	.042	TRUE	Partial mediation	13.73%
Lutropin subunit beta	0.799 (0.643–0.993)	.043	0.404 (0.225–0.729)	.003	1.031 (1.011–1.052)	.003	TRUE	Partial mediation	12.30%
Dyslexia-associated protein KIAA0319	0.799 (0.643–0.993)	.043	0.431 (0.204–0.913)	.028	1.032 (1.005–1.061)	.022	TRUE	Partial mediation	11.99%
Spliceosome RNA helicase DDX39B	0.799 (0.643–0.993)	.043	2.269 (1.020–5.049)	.045	0.975 (0.955–0.995)	.017	TRUE	Partial mediation	9.33%
RNA polymerase II subunit A C-terminal domain phosphatase SSU72	0.799 (0.643–0.993)	.043	2.336 (1.052–5.186)	.037	0.976 (0.958–0.994)	.011	TRUE	Partial mediation	9.28%
Lipase member K	0.799 (0.643–0.993)	.043	0.573 (0.348–0.942)	.028	1.037 (1.009–1.067)	.011	TRUE	Partial mediation	9.15%
Bcl10-interacting CARD protein	0.799 (0.643–0.993)	.043	2.799 (1.671–4.687)	< .001	0.980 (0.961–1.000)	.047	TRUE	Partial mediation	9.13%
Fibroblast growth factor 1	0.799 (0.643–0.993)	.043	0.521 (0.294–0.922)	.025	1.031 (1.003–1.059)	.028	TRUE	Partial mediation	8.80%
DNA-(apurinic or apyrimidinic site) lyase	0.799 (0.643–0.993)	.043	1.922 (1.184–3.119)	.008	0.971 (0.952–0.989)	.002	TRUE	Partial mediation	8.70%
Pro-FMRFamide-related neuropeptide FF	0.799 (0.643–0.993)	.043	2.463 (1.137–5.334)	.022	0.981 (0.965–0.996)	.016	TRUE	Partial mediation	7.80%
Troponin I, cardiac muscle	0.799 (0.643–0.993)	.043	1.727 (1.088–2.743)	.021	0.970 (0.947–0.994)	.015	TRUE	Partial mediation	7.39%
Inositol 1,4,5-trisphosphate receptor-interacting protein-like 1	0.799 (0.643–0.993)	.043	2.518 (1.326–4.781)	.005	0.984 (0.968–1.000)	.043	TRUE	Partial mediation	6.84%
Pulmonary surfactant-associated protein D	0.799 (0.643–0.993)	.043	2.263 (1.974–2.595)	< .001	0.984 (0.969–0.999)	.042	TRUE	Partial mediation	5.82%
Scavenger receptor class A member 3	0.799 (0.643–0.993)	.043	1.654 (1.009–2.709)	.046	0.975 (0.952–0.999)	.039	TRUE	Partial mediation	5.62%
Nucleolin	0.799 (0.643–0.993)	.043	1.499 (1.079–2.081)	.016	0.973 (0.950–0.998)	.034	TRUE	Partial mediation	4.85%

XY: total effect; XM: Step 1 mediation effect; MY: Step 2 mediation effect.

CARD = caspase recruitment domain, CI = confidence interval, DDX = dead-box RNA helicase, DNA = deoxyribonucleic acid, FMRF = phenylalanine methionine arginine phenylalanine, KIAA = Kazusa Institute – Anonymous cDNA clone, MAM = membrane associating domain, OR = odds ratio, RNA = ribonucleic acid, SSU = suppressor of SU, TBK1 = tumor necrosis factor receptor associated factor family member associated nuclear factor‑κB activator binding kinase 1.

### 3.4. Enrichment analysis

In the enrichment analysis, 59 significant pathways were identified that mediate the protective effect of elevated 25(OH)D levels on CAD incidence. To enhance the biological interpretation and prioritize the most plausible mechanisms, we categorized these pathways into 3 major functional clusters: innate immune and interferon response pathways, fibroblast growth factor receptor signaling pathways, and hormonal biosynthesis and metabolic pathways. The 3 most significant specific pathways were: severe acute respiratory syndrome coronavirus 1 (SARS-CoV-1) activates/modulates innate immune responses (Reactions found: 2/30, Entities found: 2/53, *P* value = .002), signal transducer and activator of transcription 6 (STAT6)-mediated induction of chemokines (Reactions found: 3/6, Entities found: 1/3, *P* value ≤ .001), interferon regulatory factor 3 (IRF3) mediated activation of type 1 interferon (Reactions found: 4/6, Entities found: 1/5, *P* value ≤ .001). Table 3 and Figure 4C display the significant mediating pathways, and detailed data are provided in Table S8, Supplemental Digital Content 6.

**Table 3:** Top 10 most significant pathways with mediating effects.

Pathway name	Reactions found	Reactions ratio	Entities found	Entities ratio	*P* value	Mapped proteins
SARS-CoV-1 activates/modulates innate immune responses	2/ 30	0.0020288	2/ 53	0.004476	.005179	Pulmonary surfactant-associated protein D;Serine/threonine-protein kinase TBK1
STAT6-mediated induction of chemokines	3/ 6	0.0004058	1/ 3	0.0002534	.0060629	Serine/threonine-protein kinase TBK1
IRF3 mediated activation of type 1 IFN	4/ 6	0.0004058	1/ 5	0.0004223	.0100852	Serine/threonine-protein kinase TBK1
Mineralocorticoid biosynthesis	1/ 6	0.0004058	1/ 6	0.0005067	.0120905	Lutropin subunit beta
FGFR3b ligand binding and activation	2/ 2	0.0001353	1/ 7	0.0005912	.014092	Fibroblast growth factor 1
FGFR1b ligand binding and activation	2/ 2	0.0001353	1/ 8	0.0006756	.0160895	Fibroblast growth factor 1
Defective CSF2RA causes SMDP4	1/ 1	6.763E-05	1/ 8	0.0006756	.0160895	Pulmonary surfactant-associated protein D
Defective CSF2RB causes SMDP5	1/ 1	6.763E-05	1/ 8	0.0006756	.0160895	Pulmonary surfactant-associated protein D
Orexin and neuropeptides FF and QRFP bind to their respective receptors	1/ 4	0.0002705	1/ 8	0.0006756	.0160895	Pro-FMRFamide-related neuropeptide FF
Diseases associated with surfactant metabolism	2/ 7	0.000473389	1/ 10	0.000844523	.020072924	Pulmonary surfactant-associated protein D

CSF2RA = colony-stimulating factor 2 receptor α subunit, CSF2RB = colony-stimulating factor 2 receptor β subunit, FGFR1b = fibroblast growth factor receptor 1b, FGFR3b = fibroblast growth factor receptor 3b, FMRF = phenylalanine methionine arginine phenylalanine, IFN = interferon, IRF = interferon regulatory factor, QRFP = pyroglutamylated RFamide-related peptide, SARS-CoV-1 = severe acute respiratory syndrome coronavirus 1, SMDP = sphingomyelin phosphodiesterase, STAT6 = signal transducer and activator of transcription 6, TBK1 = tumor necrosis factor receptor associated factor family member associated nuclear factor-κB activator binding kinase 1.

## 4. Discussion

CAD is one of the leading causes of death and disability worldwide, with its high morbidity and mortality rates posing a significant public health challenge. Despite considerable advances in recent years in the diagnosis and treatment of CAD (such as the application of precise diagnostic tools like coronary computed tomography angiography, the development of new drugs including glucagon-like peptide-1 receptor agonists and sodium‑glucose cotransporter-2 inhibitors, and ongoing improvements in interventional therapies and surgical techniques) the overall rates of morbidity and mortality remain high. This situation is particularly concerning in the context of an aging population and the rising prevalence of metabolic disorders.^[24–26]^ As such, identifying new risk factors that influence the onset and prognosis of CAD is of paramount importance. Among the potential candidates, vitamin D has garnered significant attention due to its broad health benefits as an essential organic compound involved in various physiological functions. Vitamin D plays a crucial role not only in maintaining calcium and phosphorus homeostasis but also in regulating immune function, exhibiting anti-inflammatory effects, and potentially improving cardiovascular health.^[27,28]^ Over 40 years ago, Scragg et al noted the seasonality of CVD and attributed it to lower levels of 25(OH)D during the winter months as a result of low sunlight exposure.^[29,30]^ Since then, the relationship between vitamin D and CAD has become a focal point of academic research. The majority of studies support the potential protective role of vitamin D in the development and progression of CAD.^[6–9]^ Nevertheless, some studies have produced conflicting findings, indicating that vitamin D supplementation therapy may have a limited effect on enhancing cardiovascular health.^[10,31,32]^ Hence, it is crucial to investigate the specific causal relationship between vitamin D and CAD. Notably, stratified MR analyses from the Emerging Risk Factors Collaboration found no evidence of a causal relationship between 25(OH)D levels and CAD.^[33]^ However, in these analyses, 25(OH)D levels were stratified into up to 10 categories, which may have led to the loss of valuable information from the original data due to the stratification of a continuous variable. Additionally, the reduction in data available in each stratum could have compromised statistical power. More importantly, stratified analysis typically only allows for the observation of risk ratios within each stratum, rather than reflecting the continuous relationship between the variable and the risk of disease. In contrast, the present study, based on large-scale GWAS data, conducted a conventional 2-sample MR analysis without stratifying the continuous 25(OH)D variable. The results suggest that higher levels of 25(OH)D are associated with a reduced risk of CAD. Further, plasma proteomics mediation analyses were used to explore potential mechanisms through which vitamin D influences CAD development, offering a comprehensive understanding of the pathways linking vitamin D deficiency to CAD. This provides new evidence supporting the recommendation for appropriate vitamin D supplementation as part of a preventive strategy for CAD patients.

Atherosclerosis is the key process in the development of CAD, and several studies have indicated that vitamin D may play a role in its regulation. Vitamin D has been shown to reduce cholesterol accumulation in macrophages and to inhibit the formation of foam cells, a marker of atherosclerosis progression, by decreasing low-density lipoprotein uptake in these cells.^[34]^ Vitamin D signaling also affects the pathophysiology of atherosclerosis by modulating the inflammatory response through decreasing the expression of tumor necrosis factor-α, IL-6, IL-1 and IL-8 in monocytes.^[35]^ In addition, vitamin D deficiency could activate NF-κB and accelerate the progression of CAD.^[36]^ As many circulating proteins are key regulators of molecular pathways, investigating their potential mediating role could provide deeper insights into the mechanisms by which vitamin D influences CAD. In this study, 19 plasma proteins were identified as mediators of the protective effect of elevated vitamin D levels against CAD. Among these, the 3 proteins with the highest mediating ratios are Serine/threonine-protein kinase TBK1, MAM domain-containing protein 2 (myeloid dendritic cell type A2 [MDCA2]), and IL-17D.

Inflammation plays a central role in the development and progression of CAD. Inflammatory factors can initiate vascular damage, promote the formation of unstable plaques, and facilitate plaque rupture by stimulating endothelial and smooth muscle cells.^[37]^ Vitamin D shows great potential in regulating inflammatory responses. Its active metabolite 25(OH)D affects multiple inflammatory signaling pathways by binding to the vitamin D receptor, inhibiting the expression of pro-inflammatory cytokines (such as IL-6 and IL-17) and promoting the secretion of anti-inflammatory factors (such as IL-10).^[38]^ In addition, vitamin D regulates the function of dendritic cells and macrophages in the innate immune system, helping to reduce excessive inflammatory responses and maintain immune balance.^[39]^ Serine/threonine-protein kinase TBK1 and IL-17D are 2 key proteins in the inflammatory response. TBK1 is a key activator of the type I interferon signaling pathway, and its overactivation can induce a chronic inflammatory state, which is closely associated with the development of atherosclerosis.^[40]^ IL-17D is a member of the IL-17 cytokine family, and its role in immune cell recruitment and pro-inflammatory responses has been extensively explored.^[41]^ High expression of IL-17D may lead to vascular inflammation, further exacerbating the progression of coronary heart disease. MDCA2 is a protein containing a mitochondrial associated membrane domain.^[42]^ Although research on its specific function is limited, existing evidence suggests that the MAM domain plays a crucial role in maintaining the interaction between mitochondria and the endoplasmic reticulum.^[43]^ Mitochondrial dysfunction and endoplasmic reticulum stress are key pathological features of CAD. The results of the present study suggest that vitamin D may exert a protective effect in CAD by regulating the expression or function of plasma proteins such as TBK1, IL-17D, and MDCA2. This regulation may help inhibit inflammatory responses, improve mitochondrial metabolism, and alleviate endoplasmic reticulum stress. However, due to the limited research on the effects of vitamin D on these specific proteins, the precise mechanisms underlying its involvement require further investigation.

In the enrichment analysis of the mediating proteins, 59 metabolic or signaling pathways were identified as being involved in the protective effect of increased 25(OH)D levels on the development of CAD. To enhance the biological interpretation and prioritize the most plausible mechanisms, we categorized these pathways into 3 major functional clusters: innate immune and interferon response pathways, fibroblast growth factor receptor signaling pathways, and hormonal biosynthesis and metabolic pathways. The most significantly enriched cluster comprised pathways related to innate immunity and interferon signaling, including STAT6-mediated induction of chemokines, IRF3-mediated activation of type 1 interferon, and SARS-CoV-1/2 modulates innate immune responses. This cluster exhibited the strongest statistical significance and biological coherence. STAT6 is a critical transcription factor that regulates immune responses and inflammation by mediating the expression of chemokines, which in turn guide the aggregation of immune cells to sites of inflammation.^[44]^ The activation of STAT6 usually interacts with the signaling pathways of cytokines such as IL-4 and IL-13.^[45]^ Vitamin D and its receptor have been reported to improve inflammation and immune responses by interfering with the STAT6-mediated pathway in a variety of diseases,^[46,47]^ which partially supports the present findings. IRF is a key transcription factor in the immune system, primarily involved in regulating the production of type 1 interferons during viral infections and inflammatory responses.^[48]^ It has been shown that vitamin D can directly affect the activation and function of IRF3. Vitamin D may enhance the production of type I interferon by increasing the phosphorylation state of IRF3 and promoting its transcriptional activity.^[49]^ This enhanced interferon response not only strengthens the body’s defence against infections but may also help mitigate the inflammatory responses associated with CAD. The recurring theme of SARS-CoV-1/2 modulation further underscores the relevance of viral response pathways, which may share common inflammatory mechanisms with CAD. In summary, the innate immune and interferon-related pathways represent the most biologically plausible and actionable mechanisms linking vitamin D to CAD risk reduction. These pathways are strongly supported by prior literature on vitamin D’s immunomodulatory effects and align with the inflammatory basis of CAD. Future studies should prioritize validating these immune-mediated mechanisms.

In the present 2-sample MR analysis, stringent criteria were employed, yielding robust evidence for the complex association between vitamin D levels and CAD. Mediation analysis further highlighted the significant role of the plasma proteome as mediators, mapping the causal pathways from increased 25(OH)D levels to protection against CAD. These insights have considerable implications for enhancing our understanding of the disease’s pathophysiology, informing preventive strategies, and potentially delaying disease progression. Nonetheless, it is vital to acknowledge several limitations in the present study. First, the genetic analyses were restricted to individuals of European ancestry, which limits the generalizability of the findings to other ethnic groups. Second, the study relied on aggregated data from publicly available sources, and as individual-level clinical data were not accessible, detailed population stratification analyses could not be performed. Third, experimental validation of the identified mediating proteins and the underlying mechanisms linking vitamin D to CAD was not conducted. Future research should aim to address these gaps by confirming this causal relationship through large, prospective, multicenter cohort studies and by conducting experimental studies to verify the mechanistic pathways identified in the present analysis.

## 5. Conclusion

In the present study, it was found that elevated 25(OH)D levels significantly reduce the risk of CAD. Notably, the underlying mechanisms through which increased 25(OH)D levels confer protection against the development of CAD were identified, offering novel insights and potential therapeutic targets for the prevention and treatment of CAD.

## Acknowledgments

The authors thank Yuying Li Ph.D from the First Hospital of Jilin University for great assisting the preparation of this paper. This work was supported by the Scientific Research Project of Hunan Provincial Health Commission (Grant No. D202303018575), and the Jiont research foundation organized by Hunan University of Chinese Medicine and Yiyang Central Hospital (Grant No. 2022XYLH106). The funders had no role in study design, data collection and analysis, decision to publish, or preparation of the paper.

## Author contributions

**Conceptualization:** Yanqiong Xue.

**Formal analysis:** Shunhua Cao.

**Methodology:** Yanqiong Xue.

**Writing – original draft:** Yanqiong Xue.

**Writing – review & editing:** Linxia Zhang, Jiaquan Chen, Aili Ning.
















